# Diversity in Sociodemographic and Linguistic landscape: Perspectives from Chinese American, Mexican American, and Indian Cohorts

**DOI:** 10.1002/alz.090672

**Published:** 2025-01-03

**Authors:** Boon Lead Tee, Suvarna Alladi, Nithin Thanissery, Ganesh M. Babulal, Sid E. O'Bryant, Melissa Petersen, Isabel Elaine Allen

**Affiliations:** ^1^ Global Brain Health Institute/ University of California, San Francisco, San Francisco USA; ^2^ Memory and Aging Center, UCSF Weill Institute for Neurosciences, University of California, San Francisco, San Francisco, CA USA; ^3^ National Institute of Mental Health and Neurosciences [NIMHANS], Bengaluru, Karnataka India; ^4^ National Institute of Mental Health and Neuro Sciences, Bengaluru, Karnataka India; ^5^ Washington University School of Medicine, St. Louis, MO USA; ^6^ Knight Alzheimer Disease Research Center, St. Louis, MO USA; ^7^ University of North Texas Health Science Center, Fort Worth, TX USA; ^8^ Department of Epidemiology and Biostatistics, University of California, San Francisco, San Francisco, CA USA; ^9^ Global Brain Health Institute, University of California San Francisco, San Francisco, CA USA

## Abstract

**Background:**

Nearly half the world’s population is bi‐ or multilingual, where diverse interpretations of bilingualism with varying associated sociodemographic factors may lead to inconsistent conclusions in bilingualism and cognitive research and thus our study aimed to further examine this.

**Method:**

We examined the sociodemographic features and bilingualism phenotypes among monolingual and bilingual individuals across three cohorts: Chinese American (CA) from UCSF Memory and Aging Center (n = 517), Mexican American (MA) from HABS‐HD project (n = 1,166), and Indians (ID) from National Institute of Mental Health and Neuroscience (NIMHANS) (n = 1,233), encompassing individuals with normal cognition, mild cognitive impairment (MCI), and Alzheimer’s dementia (AD). We compared group differences between monolinguals and bilinguals via Student’s t‐test for continuous variables and with Pearson’s Chi‐squared test for categorical variables. Group comparison of bilingualism phenotypes across cohort were analyzed using ANOVAs for continuous variables (with Bonferroni correction for multiple comparisons).

**Result:**

Educational disparities were significant between monolinguals and bilinguals across all cohorts (all p<0.001), with the exception of MCI (monolinguals: 8 *±*1.8 years, bilinguals: 9.6 *±*6.3 years, p = 0.104) and AD groups (monolinguals: 4.4 *±* 6.7 years, bilinguals: 5.5 *±* 6.1 years, p = 0.722) at NIMHANS. Notably, a greater prevalence of illiteracy among monolinguals emerged in the NIMHANS (monolinguals: 10%, bilinguals: 2%, p<0.001) and HABS‐HD cohorts (monolinguals: 4%, bilinguals: 2%, p = 0.034), a trend absent in the UCSF group (monolinguals: 0%, bilinguals: 0%). In HABS‐HD, monolinguals with normal cognition (p<0.001) or MCI (p = 0.019) resided in neighborhoods with greater deprivation, whereas those in NIMHANS control group indicated lower socioeconomic status in monolinguals (p<0.001), a distinction not found in UCSF. Additionally, bilinguals in the UCSF CA and NIMHANS ID cohorts were observed to speak a greater number of languages (p<0.001) that largely belonging to different language families. Naturally, when examining the maximum spoken language distance of bilingual participants, UCSF CA and NIMHANS ID bilinguals had significantly higher language distances compared to bilinguals in the HABS‐HD cohort (p<0.001).

**Conclusion:**

These findings emphasize the diverse bilingualism phenotypes and associated sociodemographic factors within distinct populations and underscore the imperative need for a comprehensive characterization of language history in bilingualism research.

